# Effects of Conjugated Linoleic Acid Supplementation on the Expression Profile of miRNAs in Porcine Adipose Tissue

**DOI:** 10.3390/genes8100271

**Published:** 2017-10-13

**Authors:** Qi Wang, Renli Qi, Hong Liu, Jing Wang, Wenming Huang, Feiyun Yang, Jinxiu Huang

**Affiliations:** 1 Chongqing Academy of Animal Sciences, Chongqing 402460, China; wangq0418@126.com (Q.W.); qirenli1982@163.com (R.Q.); dkbshliuhong@sina.com (H.L.); wj57482199@163.com (J.W.); 2 Key Laboratory of Pig Industry Sciences, Ministry of Agriculture; Chongqing Key Laboratory of Pig Industry Sciences, Chongqing 402460, China; 3 The Department of Animal Husbandry, Rongchang Campus, Southwest University, Rongchang, Chongqing 402460, China; hwmyy@126.com

**Keywords:** miRNA, conjugated linoleic acids, pig, adipose tissue, differential expression

## Abstract

Conjugated linoleic acids (CLAs) play a major role in adipocyte differentiation and lipid metabolism in animals. MicroRNAs (miRNAs) appear to be involved in many biological processes in adipose tissue. However, the specific influence on miRNAs by CLA supplementation in porcine adipose tissue remains unclear. Thus, we continuously added 1.5% CLA to the pig diet from the embryo stage to the finishing period and conducted a high-throughput sequencing approach to analyse the changes in adipose tissue miRNAs. We identified 283 known porcine miRNAs, and 14 miRNAs were differentially expressed in response to CLA treatment. A Kyoto Encyclopedia of Genes and Genomes (KEGG) analysis showed that the targets of the 14 differentially expressed miRNAs were involved in the Wnt signalling pathway. The CLA treatment downregulated the gene expression of PPARγ, C/EBPα, FAS, and FATP1 in both subcutaneous and abdominal fat tissues; the analysis showed that ssc-miR-21 expression was significantly correlated with PPARγ expression (*p* < 0.05), and speculated that ssc-miR-21 might influence adipogenesis through PPARγ. In conclusion, our study analysed the miRNA profiles in porcine adipose tissues by CLA treatment, and demonstrated that miRNAs are important regulators of fat lipogenesis. This study provides valuable information for the molecular regulatory mechanism of CLA on adipose tissue.

## 1. Introduction

Conjugated linoleic acid (CLA) is an octadecadienoic acid containing a conjugated double bond and is often found in fats from ruminants. CLA has several physiological functions, such as improving animal immunity, anti-atherosclerosis, anti-cancer, inhibition of oxidative stress, and reduced body fat deposition [1,2,3]. The two major isomers of CLA are cis-9,trans-11 CLA (CLA-c9,t11), and trans-10,cis-12 CLA (CLA-t10,c12), which have different biological properties.

Adipose tissue is the most important tissue for energy storage, and it is crucial for lipid metabolism [4]. Several studies have shown that CLA plays an important role in reducing adipose fat in animals [5]. For example, 0.2% and 0.6% CLA supplementation resulted in a greater body fat reduction compared to a control group in mice [6]. Ramiah et al. reported that feeding CLA to broiler chickens downregulated the expression of peroxisome proliferator activated receptor γ (PPARγ) and adipocyte protein 2 (aP2) in abdominal fat and reduced abdominal adipocyte size, effects which decreased the capacity to store fat [7]. Research on humans also indicated that CLA significantly reduced body fat mass or abdominal fat in obese humans [8,9].

MicroRNAs (miRNAs) are a class of small, non-coding RNAs that modulate gene expression at the post-transcriptional level in animals and plants. They are involved in various biological processes, including cell growth, differentiation, apoptosis, and metabolism [10,11,12]. miRNAs can influence the metabolism of adipose tissue, and Meng et al. reported that metabolic syndrome (MetS) changed the miRNA expression profile of porcine adipose tissue mesenchymal stem cell (MSCs)-derived extracellular vesicles (EVs), and that 14 and eight distinct miRNAs were enriched in Lean-EVs and MetS-EVs, respectively [13]. Since miRNA levels reflected components of the metabolic syndrome, researchers considered miRNA to have potential to be novel biomarkers for this complex syndrome [13,14]. Moreover, a growing number of studies have indicated that changes in the diet of animals could influence the expression of miRNAs. A study by Li et al. indicated that eight core miRNAs in bovine mammary glands were differentially expressed by treatments supplemented with either linseed oil or safflower oil [15]. Sun et al. found that twenty-five miRNAs were differentially expressed between a normal protein diet or a low protein diet in piglets, and that miR-19b was predicted to be involved in urea cycle metabolism by targeting Sirtuin 5 (SIRT5) [16]. Feeding with an obesogenic diet will alter the expression of specific miRNAs related to lipid metabolism. For example, the expression of miRNA-122 was decreased by a high-cholesterol diet in minipigs; miR-143 expression was significantly increased on the high fat diet [17,18,19]. In addition, miR-143 expression levels were correlated with PPARγ and aP2, genes which regulate adipocyte differentiation and lipid metabolism.

CLA treatment altered the expression of adipose-related miRNAs (miR-143, miR-103, miR-107, miR-221, and miR-222) in the adipose tissue of mice [20]. In our previous studies, we found that 1.5% CLA was the most appropriate dose for improving the carcass traits and meat quality of pigs, and CLA significantly altered the expression of miR-27, miR-143, and adipocyte differentiation genes in adipose tissue of growing pigs [21]. In the present study, we firstly compared the differentially expressed (DE) miRNAs following 1.5% CLA supplementation from the embryo stage to the finishing period and explored which miRNAs were implicated in the process with the aim of elucidating the molecular mechanisms of CLA on adipose development.

## 2. Materials and Methods

### 2.1. Ethics Statement

All research involving animals was performed according to the Regulations for the Administration of Affairs Concerning Experimental Animals (Ministry of Science and Technology, China; revised in June 2004) and adhered to the Reporting Guidelines for Randomized Controlled Trials in Livestock and Food Safety (REFLECT). The Institute ethics committee of the Chongqing Academy of Animal Science (Chongqing, China) reviewed that relevant ethical issues in this study were considered (permit number xky-20150113).

### 2.2. Animals, Diet, and Sample Collection

Rongchang pigs are used in this study, which are a typical representative of indigenous pigs from southwestern China and are characterized by better meat quality and high body fat mass. All healthy pigs were fed in a standard experimental piggery of the Chongqing Academy of Animal Sciences. Purebred pregnant Rongchang sows and their piglets were randomly assigned into control or CLA groups (eight sows/group). The pregnant sows in the CLA group were fed a 1.5% CLA diet from the start of pregnancy until weaning, and 1.5% CLA was added to the diet of their piglets from weaning to the finishing period. The diet of pregnant sows and their piglets is listed in Table S1 and Table 1. The control group was fed a corn–soybean meal basal diet, while the CLA group was fed a diet of 1.5% CLA (purity 61.2%; AuHai Biotech Co. Ltd., Qingdao, China). CLA was used as a substitute for soybean oil in the basal diet.

Groups of twelve pigs were respectively weighted and then slaughtered at 30, 90, and 240 days old for tissue sampling. The dorsal subcutaneous fat tissues (SF), which are located near the left side of the scapula, were collected at each period, while abdominal fat tissues(AF) were collected only at day 240. All samples were rinsed with phosphate buffered saline (PBS) and quickly frozen in liquid nitrogen.

### 2.3. RNA Extraction

Total RNA was extracted using a Trizol reagent (Invitrogen, California, USA) as follows. Tissue samples in 1 mL of Trizol reagent per 50 to 100 mg of tissue were homogenized. The homogenate was separated in aqueous and organic phases by the addition of 200 μL chloroform followed by centrifugation at 12,000 *g* for 15 min at 4 °C. Five hundred microliters (500 μL) of the upper layer aqueous phases containing RNA were drawn into a new Eppendorf tube. RNA was precipitated by the addition of 500 μL isopropanol and centrifugation at 12,000 *g* for 10 min at 4 °C. After discarding the supernatant, the resulting RNA was washed in 75% ethanol and solubilized in diethyl pyrocarbonate-treated water. After elution, the quality analysis of total RNA was performed by using the NanoDrop instrument (Thermo Fisher, Waltham, MA, USA). Equal quantities of RNA from the adipose tissues of three individual pigs were pooled from the same group (Table S2). For subcutaneous fat tissues, two libraries per diet treatment (Control or CLA) were made for the respective growing period (30, 90, and 240 days old), and it appears that only tissues from the final period (240 days old) were used to construct two libraries (Control or CLA) for abdominal fat tissues.

### 2.4. Small RNA Library Construction and Sequencing

The 16 pooled samples were prepared to construct complementary DNA (cDNA) libraries following the steps below. Polyacrylamide gel electrophoresis (PAGE) was used to isolate the 18–30 nt small RNA(sRNA) segments. Then, the purified sRNA was ligated with 5′ and 3′ adaptors using T4 RNA ligase, respectively. Reverse transcription followed by PCR was used to create cDNA constructs based on the adaptor-ligated sRNA. The amplified cDNA constructs were recycled and purified from the PAGE gel; an Agilent 2100 Bioanalyzer (Agilent, Palo Alto, CA, USA) and an ABI StepOnePlus Real-Time PCR System (ABI, Carlsbad, CA, USA) were used to check the quality and yield. Finally, the resultant 16 cDNA libraries were deep sequenced on an Illumina HiSeq 2000 platform (Illumina, SanDiego, CA, USA) according to the manufacturer’s instructions by the BGI Corporation.

### 2.5. Data Analysis and miRNA Annotation

The data that were low quality reads, including reads with 5′ adaptor pollution or poly (A) stretches, reads without 3′ adaptors, and reads shorter than 18 nt, were filtered from the file. Reads that passed the filtering step were annotated and classified by comparing the sequences with the non-coding RNAs (rRNA, tRNA, scRNA, snRNA, and snoRNA) databases in GenBank (http://www.ncbi.nlm.nih.gov/). Known porcine miRNAs were identified from miRBase (version 21.0). The small RNA (sRNA) that mapped to antisense exons, introns, or intergenic regions of the genome and do not map to porcine known sRNA were predicted to be novel miRNAs. miRDeep2 software (Max Delbrück Center, Berlin-Buch, Germany) was used to predict novel porcine miRNAs by exploring their secondary structure, the Dicer cleavage site, and the minimum free energy of the unannotated small RNA reads. Significantly DE miRNAs were defined as having log2 fold change (FC) >1 or <−1, *p*-value < 0.05 and false discovery rate (FDR) <0.05. All the generated RNA-seq data can be found in the supplemental file S1.

### 2.6. Functional Analysis of Significantly Differentially Expressed miRNAs

The target genes of 14 significantly DE miRNAs were predicted using miRanda and TargetScan softwares. A KEGG pathway analysis identified the main biochemical and signal transduction pathways for the candidate target genes. Significantly enriched pathways were defined as having a *p*-value < 0.05. Gene ontology (GO) terms were also assigned to the predicted target genes, and an enrichment analysis was performed using GOseq and topGO to determine their likely biological functions.

### 2.7. miRNA Validation and Gene Expression Analysis

Six miRNAs from 14 DE miRNAs were selected to validate the sRNA sequencing results by performing real-time quantitative PCR (qPCR). RNA was extracted using a MiniBEST Universal RNA Extraction Kit (TaKaRa, Beijing, China) according to the manufacturer’s protocol, and qPCR was performed on a pool of RNA samples from the adipose tissues of three individual pigs from the same group. Otherwise, we evaluated the expression of adipogenic transcription factors and adipocyte-related genes of interest. The miRNA and mRNA expression was assessed by the SYBR PrimeScript miRNA RT-PCR Kit or the SYBRPremix Ex TaqII kit (TaKaRa, Beijing, China) following the manufacturer’s instructions, respectively. The reactions were run in triplicate by a QuantStudio 6 Flex RT-PCR system (Life Technologies, Carlsbad, CA, USA), and the data were analysed by using the 2^−△△CT^ method. All of the primers used in this study are listed in Table S3. Porcine U6 snRNA and GAPDH were used as reference genes for miRNA and mRNA expression analysis, respectively.

### 2.8. Statistical Analysis

The significance of differences between the control group and the CLA group was determined via one-way analysis of variance (ANOVA). Linear relationships between the key variables were determined using Pearson’s correlation coefficients. SPSS for Windows version 19 (SPSS, Chicago, IL, USA) was used for the analysis.

## 3. Results

### 3.1. Overview of the Deep sRNA Libraries

The pooled adipose tissue libraries were sequenced on a HiSeq platform. Fifteen libraries out of 16 were successfully sequenced. The raw reads of library (90-SF4) were less than 10,000, and this library was thus discarded for further processing. The sRNA sequences were searched against the sequences in the GenBank and Rfam3 databases, and the annotated results are summarized in Figure S1. After removing contaminated data, more than 79 million clean read counts were obtained. Table S4 summarizes the information of length distribution; the majority of the reads were 22 nt in length in both of the two adipose tissues.

### 3.2. Identification of Known miRNAs and New miRNAs in Pigs

The identified miRNA sequences were annotated by matching them against the known miRNA sequences in miRBase 21.0. As a result, 283 known porcine miRNAs and 90 new miRNAs were identified in 15 libraries. The mature and precursor sequence, genome loci, dominant arm, hairpin structure, and the minimum free energy (MEF) of new miRNAs are listed in Table S5; 36.7% (33/90) of new miRNAs are 5p-arm, and 63.2% (67/90) are 3p-arm.

**Figure 1:**
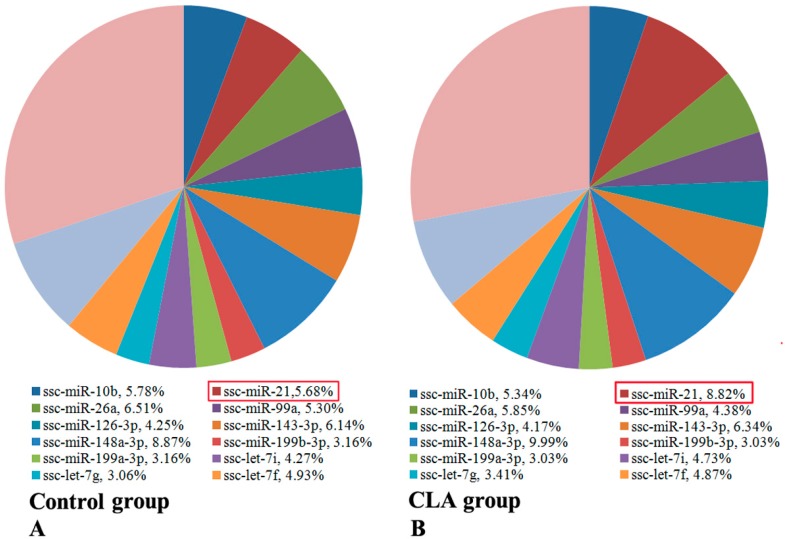
Thirteen known miRNAs highly expressed in both subcutaneous and abdominal adipose tissues from deep sequencing. (**A**) The read counts of miRNAs were collected at each period of subcutaneous adipose tissues and at day 240 of abdominal adipose tissues from the control group; (**B**) The read counts of miRNAs were collected at each period of subcutaneous adipose tissues and at day 240 of abdominal adipose tissues from the conjugated linoleic acid (CLA) treatment group.

In the known porcine miRNAs, a large number of miRNAs were located on Chr X (32 miRNAs), Chr 1 (24 miRNAs), and Chr 2 (21 miRNAs), while only 12 miRNAs in total were mapped to Chr 8, 15, and 16 (Table S6). We found that 13 miRNAs (ssc-miR-10b, -21, -26a, -99a, -126-3p, -143-3p, -148a-3p, -199a-3p, -199b-3p, -let-7i, -let-7g, -let-7f, and -let-7a) were highly expressed in both subcutaneous and abdominal adipose tissues from deep sequencing. These 13 miRNAs accounted for 69.91% and 72.01% of all of the known miRNAs in the control group and the CLA treatment group, respectively. The remaining miRNAs were expressed at lower levels. Among 13 miRNAs, ssc-miR-21 expression was significantly upregulated following treatment with CLA (Figure 1 and Table S7).

### 3.3. DifferentiallyExpressed miRNAs in Response to CLA Supplementation

**Figure 2:**
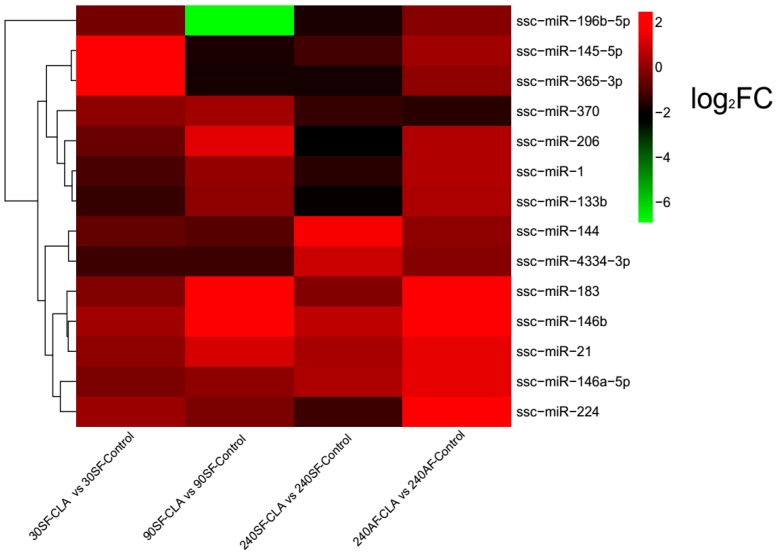
Hierarchical clustering analysis for 14 differentially expressed miRNAs for CLA group versus control group from sequencing. Abbreviations: 30SF, 90SF, and 240SF: dorsal subcutaneous adipose tissue at 30, 90, and 240 days old, respectively; 240AF: abdominal adipose tissues at 240 days old. Log_2_FC: log2 fold change.

Our study showed that CLA supplementation significantly increased the porcine body weight at 90 and 240 days old, and decreased the abdominal fat weight and fat thickness of pigs (Table S8). Based on the criterion of log_2_FC >1 or <–1, *p*-value < 0.05, and FDR < 0.05, five, three, and three DE miRNAs in subcutaneous adipose tissues were found at 30, 90, and 240 days old, respectively, while six DE miRNAs were found in abdominal adipose tissues at 240 days old. Three miRNAs appeared twice among them, thus a total of 14 miRNAs (ssc-miR-1, -21, -133b, -144, -145-5p, -146a-5p, -146b, -183, -196b-5p, -206, -224, -365-3p, -370, and -4334-3p) were detected to differentially express in response to 1.5% CLA treatment (Table 2, Figure 2, and Table S9). Ssc-miR-21 and ssc-miR-146b were expressed differentially in both adipose tissues. Diets with 1.5% CLA caused the expression of ssc-miR-21 and ssc-miR-146b to be upregulated at 30, 90, and 240 days old in subcutaneous adipose tissues, and at 240 days old in abdominal adipose tissues. Moreover, we also detected three new miRNAs (new-miR-9, new-miR-64, and new-miR-80) differentially expressed in response to 1.5% CLA treatment, and new-miR-9 was a differential expression miRNA in both of the two adipose tissues (Table S10).

### 3.4. Target Prediction of Differentially Expressed miRNAs and Functional Analysis

**Figure 3:**
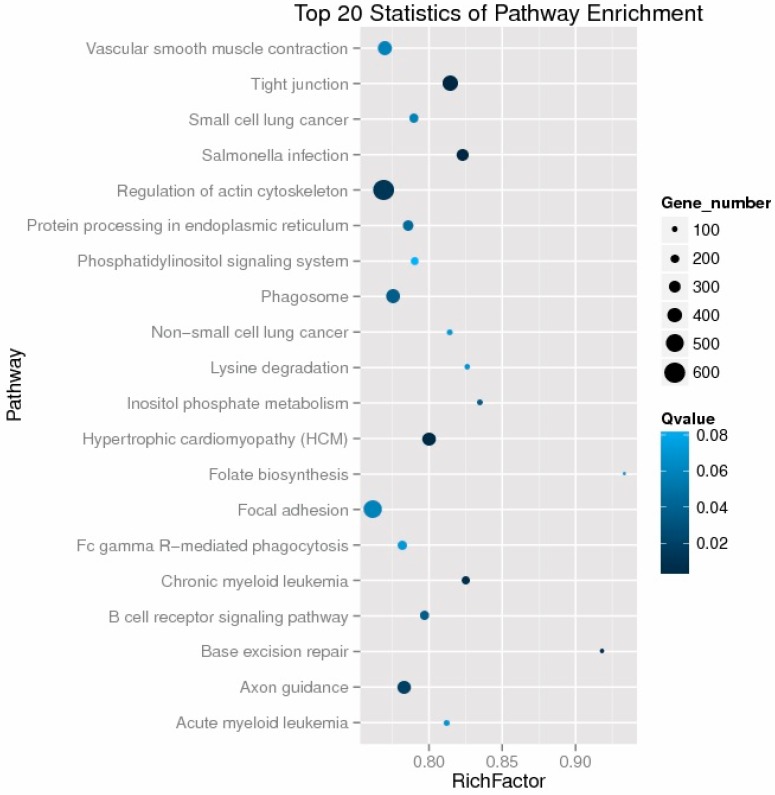
The top 20 KEGG pathways enriched for targets of the 14 differentially-expressed miRNAs.

To better understand the biologicalfunction of 14 DE miRNAs, we used miRanda and TargetScan software to predict the target genes and extracted the intersection of the two methods as a prediction result. Subsequent KEGG Orthology analysis was performed to reveal the main pathways in which the target genes may be involved; 49 significantly enriched KEGG pathways were identified (*p* < 0.05) (Table S11). The top 20 enriched pathways for the target genes of the 14 DE miRNAs are shown in Figure 3. In the 49 significant KEGG pathways, seven pathways were associated with cancer, including pathways in cancer, small cell lung cancer, prostate cancer, pancreatic cancer, colorectal cancer, thyroid cancer, and non-small cell lung cancer. The Notch signalling and Wnt signalling pathways are involved in adipogenesis, and the adipocytokine signalling pathway is correlated with the production of leptin and adiponectin [22,23,24]. Another two pathways, the inositol phosphate metabolism pathway and the phosphatidylinositol signalling system, are important for lipid metabolism [25,26]. GOseq and topGO were used to describe the enrichment level for each GO term. The analysis showed that the most significantly enriched terms were cellular process, cell/cell part, and binding under the biological process, cellular component, and molecular function categories, respectively (Figure S2).

### 3.5. Validation of Sequencing Data

**Figure 4:**
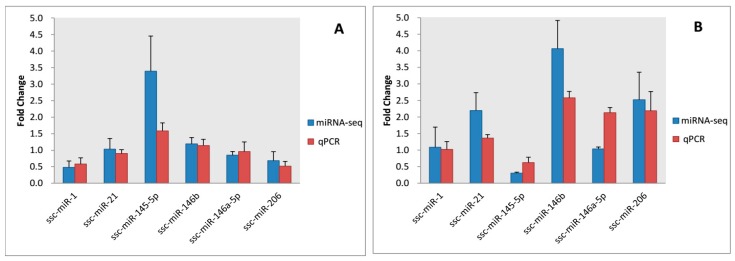

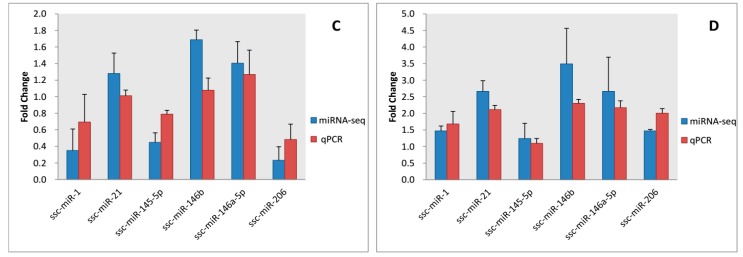
Six differentially-expressed (DE) miRNAs validated by qPCR. (**A**) dorsal subcutaneous adipose tissue at 30 days old; (**B**) dorsal subcutaneous adipose tissue at 90 days old; (**C**) dorsal subcutaneous adipose tissue at 240 days old; and (**D**) abdominal adipose tissue at 240 days old. Four, three, four, and four pool RNA samples were used for miRNA-sequencing (miRNA-seq) or qPCR in (**A**–**D**).

Six DE miRNAs (ssc-miR-1, -21, 145-5p, -146b, -146a-5p, and -206) were selected for qPCR to validate the RNA sequencing results. The Pearson’s correlation coefficients for the expression results were 0.902 (*p* = 0.014), 0.775 (*p* = 0.071), and 0.904 (*p* = 0.013) at 30, 90, and 240 days old in subcutaneous adipose, respectively, and 0.799 (*p* = 0.057) at 240 days old in abdominal adipose (Figure 4). The results confirmed positive correlations between the qPCR results and the miRNA-sequencing data.

### 3.6. Correlations between miRNAs and Adipogenic Transcription Factors or Adipocyte Genes with CLA

**Figure 5:**
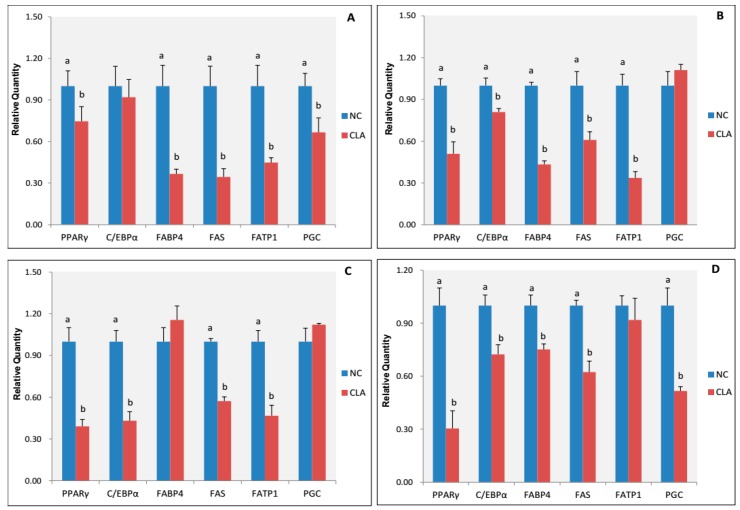
Expression level of adipogenic transcription factors and adipocyte genes in subcutaneous adipose tissues and abdominal adipose tissues of pigs. NC: control group; CLA: CLA treatment group. (**A**) dorsal subcutaneous adipose tissue at 30 days old; (**B**) dorsal subcutaneous adipose tissue at 90 days old; (**C**) dorsal subcutaneous adipose tissue at 240 days old; (**D**) abdominal adipose tissue at 240 days old. Upper letters (a, b) on bars denote significantly different expression levels in the same gene (*p* < 0.05).

To reveal the effect of continuous dietary addition of 1.5% CLA on key adipogenic transcription factors and adipocyte genes expression, we used qPCR to analyse the expression levels of adipocyte-related genes, such as PPARγ, CCAAT/enhancer binding protein alpha (C/EBPα), fatty acid binding protein 4 (FABP1), fatty acid transport protein 1 (FATP1), fatty acid synthetase (FAS), and peroxisome proliferative activated receptor-γ coactivator 1 alpha (PGC-1α). In subcutaneous adipose tissues, the expression of PPARγ and C/EBPα in the CLA group declined with age. With the exception of PGC-1α and FABP4, CLA treatment decreased the expression of PPARγ, C/EBPα, FAS, and FATP1 in both subcutaneous and abdominal adipose tissues (Figure 5).

We further analysed correlations between the expression of DE miRNAs (ssc-miR-21 and ssc-miR-146b) and the adipocyte phenotype. miR-21 expression significantly correlated with PPARγ expression (*r* = −0.959, *p* < 0.05). However, no significant correlation was observed between the selected miRNAs and other adipocyte genes (Table 3).

## 4. Discussion

CLA has an effect on the lipid profile, hormone secretion, and enzymes activity in animals, and changes the expression of related transcription factors and genes. As an important regulatory molecule, miRNA expression is also influenced by the changes of CLA. In this study, we examined the effect of CLA treatment on the miRNome expression in swine adipose tissue by using high-throughput sequencing. Fourteen miRNAs in porcine adipose tissues were differentially expressed in response to a continuous addition of dietary 1.5% CLA. Among them, ten DE miRNAs were found at three growth stages in subcutaneous adipose tissues, while six miRNAs were differentially expressed in abdominal adipose tissues at 240 days old.

Among these 14 DE miRNAs, miR-21 and miR-146b were identified in both adipose tissues and we speculated that they played a crucial role in adipogenesis by CLA treatment. It has been demonstrated that miR-21 is a representative miRNA that is functionally involved in lipid metabolism and adipogenesis [27]. In high-fat-diet-fed mice, the expression of miR-21 was decreased in the liver compared with chow-fed mice [28]. An overexpression of miR-21 significantly blocked stearic acid-induced intracellular lipid accumulation by targeting fatty acid-binding protein 7 (FABP7). In the miR-21^−/−^ mice, the gene expression profiles showed that groups of lipid metabolism genes were changed, including PPARα, which was identified as a direct target of miR-21 [29]. Several studies in vitro have shown that miR-21 was also involved in adipogenesis. Kang et al. found that miR-21 significantly promoted adipocyte differentiation by increasing the expression of adiponectin and decreasing activator protein 1 (AP-1) level in 3T3-L1 adipocytes [30]. In human adipose tissue-derived mesenchymal stem cells (hASCs), miR-21 enhanced adipogenesis by modulating the transforming growth factor beta (TGF-β) signalling pathway, and an overexpression of miR-21 decreased the cell proliferation of hASCs by targeting the signal transducer and activator of transcription 3 (STAT3) [31]. In the present study, we found a significant negative correlation between the expression of miR-21 and PPARγ in adipose tissues, suggesting that the greater levels of miR-21 induced by CLA treatment resulted in decreased PPARγ expression. On the other hand, another DE miRNA, miR-146b, was highly expressed in mature adipocytes. miR-146b directly bound to SIRT1, which plays a key role in metabolic homeostasis and promotes fat mobilization in white adipose tissue. The miR-146b/SIRT1 axis mediates adipogenesis through increased acetylation of forkhead box O1 (FOXO1) in 3T3-L1 cells [32]. A study by Chen et al. reported that miR-146b could inhibit the proliferation of human visceral preadipocytes and promote cell differentiation by inhibiting the expression of Kruppel-like transcription factor7 (KLF7). Moreover, miR-146b was also confirmed to be an important mediator in adipose tissue inflammation [33,34]. In the current study, we demonstrated that miR-146b expression was significantly upregulated by CLA treatment, but miR-146b showed no significant correlation with the selected adipocyte genes, suggesting that miR-146b possibly regulates lipogenesis by impacting other fat-related genes in porcine adipose tissue.

Among another 12 DE miRNAs, three miRNAs (miR-1, -133b, and -206) are defined as myogenic miRNAs [35,36]. miR-145-5p (miR-145), miR-146a-5p, miR-183, miR-196b-5p, and miR-224 were associated with adipogenesis in mammals. miR-145 inhibits adipogenesis by targeting insulin receptor substrate 1 (*IRS1*), while miR-224 negatively regulates early adipogenesis via early growth response 2 (*EGR2*) [37,38]. In primary porcine adipocytes, miR-146a-5p inhibited TNFα-induced adipogenesis by controlling insulin receptor (IR) expression [39]. Furthermore, miR-183 promoted differentiation and adipogenesis by inactivating the Wnt/β-catenin pathway and targeting low-density lipoprotein receptor-related protein 6 (LRP6) in 3T3-L1 cells [40]. A study by Liu et al. found that miR-196b-5p could influence porcine adipogenesis in muscle through the adipocytokine signalling pathway [41]. miR-370 and miR-144 have been shown to be involved in lipid metabolism. Iliopoulos et al. observed that miR-370 directly targeted the 3′-UTR of carnitine palmitoyltransferase 1α (Cpt1α) and decreased the rate of fatty-acid β-oxidation; miR-144 could bind the 3′-UTR of Elongation of very long chain fatty acids protein 6 (ELOVL6) to control its expression in duck liver [42,43]. The other two miRNAs (miR-365-3p and miR-4334-3p) are less studied. miR-365-3p was found to have an effect on the expression of the placenta-expressed transcript 1 (*PLET1*) gene, which is important for placental development in pigs [44]. There is no research on miR-4334-3p to date.

To better understand the biological functions of the predicted target genes, 49 significantly enriched pathways were identified by a KEGG Orthology analysis (*p* < 0.05). In our study, 237 putative target genes that are regulated by 14 DE miRNAs were found to be involved in the Wnt signalling pathway (Figure S3), which is one of the most important signalling pathways controlling lipogenesis and adipogenesis. PPARγ and C/EBPα are the key adipogenic transcription factors that trigger adipocyte differentiation. Previous studies reported that Wnt could block PPARγ and C/EBPα expression to inhibit adipogenesis [45,46]. Yeganeh et al. showed that CLA-t10,c12 treatment increased the levels of β-catenin and its activity in 3T3-L1 adipocytes. β-catenin binds to PPARγ, and inhibits its activity to prevent the progression of adipogenesis [47,48]. These researches confirmed that CLA inhibited adipocyte adipogenesis via Wnt/β-catenin signalling. In our present study, CLA treatment decreased the levels of adipogenic genes such as PPARγ and C/EBPα. Thus, we speculate that a continuous dietary addition of 1.5% CLA prevented adipogenesis in porcine adipose tissues by regulating the Wnt signalling pathway.

In conclusion, dietary supplementation with 1.5% CLA altered the expression profile of miRNAs in porcine adipose tissue. Fourteen miRNAs were significantly differentially expressed in response to CLA treatment. These results indicated that miRNAs could be important regulators of porcine adipose lipogenesis, and provide knowledge and ideas for the future study of the molecular regulatory mechanism of miRNAs by CLA in porcine adipose tissues.

## Figures and Tables

**Table 1:** Composition of the basal diets for growing pigs at different periods (air-dry basis, %).

Items	6–15 kg	15–30 kg	30–60 kg	60–90 kg
Ingredients (%)				
Corn	57.20	60.00	70.30	72.60
Soybean meal	-	24.00	14.00	11.00
Extruded soybean meal	12.95	-	-	-
Extruded soybean	12.21	-	-	-
Fishmeal	5.30	-	-	-
Wheat bran	-	11.35	11.50	12.50
Whey powder	5.83	-	-	-
Soybean oil ^a^	3.95	2.00	2.00	2.00
Limestone	0.90	0.56	0.71	0.71
Calcium hydrogen phosphate	0.47	1.00	0.50	0.10
NaCl	0.25	0.30	0.30	0.30
Lysine	0.14	0.10	0.00	0.10
Premix ^b^	0.80	0.69	0.69	0.69
Total	100.00	100.00	100.00	100.00
Nutrient levels ^c^				
DE (MJ/kg)	14.96	13.75	13.84	13.83
CP (%) ^b^	19.53	18.83	15.95	13.95
Ca (%) ^b^	0.81	0.82	0.72	0.61
TP (%)	0.75	0.67	0.54	0.47
AP (%)	0.42	0.37	0.29	0.22
DLys (%)	1.22	0.85	0.71	0.49
DMet (%)	0.27	0.25	0.21	0.19
DMet+DCys (%)	0.69	0.53	0.43	0.39
DThr (%)	0.78	0.55	0.43	0.37
DTrp (%)	0.23	0.19	0.14	0.12

^a^ 1.5% CLA as used as a substitute for soybean oil in the basal diet of the CLA group. ^b^ Provided per kilogram of diet: Cu (CuSO_4_·5H_2_O) 80 mg, Fe (FeSO_4_·7H_2_O) 100 mg, Zn (ZnSO_4_·7H_2_O) 100 mg, Mn (MnSO_4_·H_2_O) 40 mg, Se (Na_2_SeO_3_) 0.3 mg, I (KI) 0.3 mg, VA 1 750 IU, VD3 200 IU, VE 11 IU, VK3 0.5 mg, niacin 20 mg, pantothenic acid 9 mg, folic acid 0.3 mg, VB1 1 mg, VB2 3 mg, VB6 1.5 mg, VB12 15 μg, biotin 0.05 mg, choline chloride 1.0 g, phytase 0.1 mg, and anti-oxidant 0.5 mg. ^c^ Measured value. DE: Digestable energy, CP: Crude protein, TP: Total phosphorus, AP: Available phosphorus, DLys: Digestible Lysine, DMet: Digestible Methionine, DThr: Digestible Threonine, DTrp: Digestible Tryptophan.

**Table 2:** Differentially expressed miRNAs in response to 1.5% CLA treatment from deep sequencing.

miRNA	Log_2_FC ^b^	*p*-Value	FDR ^c^
**Back fat/Day 30**			
ssc-miR-1	–1.045	0.000	0.028
ssc-miR-133b	–1.292	0.001	0.034
ssc-miR-145-5p	1.763	0.000	0.000
ssc-miR-365-3p	1.798	0.000	0.000
ssc-miR-4334-3p	–1.247	0.000	0.019
**Back fat/Day 90**			
ssc-miR-21 ^a^	1.134	0.001	0.046
ssc-miR-146b ^a^	2.023	0.000	0.002
ssc-miR-196b-5p	–6.897	0.000	0.000
**Back fat/Day 240**			
ssc-miR-133b	–1.998	0.000	0.043
ssc-miR-144	1.686	0.000	0.009
ssc-miR-206	–2.114	0.000	0.010
**Abdominal fat/Day 240**			
ssc-miR-21 ^a^	1.414	0.000	0.000
ssc-miR-146b ^a^	1.804	0.000	0.000
ssc-miR-146a-5p	1.413	0.000	0.000
ssc-miR-183	2.127	0.000	0.003
ssc-miR-224	2.496	0.000	0.019
ssc-miR-370	–1.530	0.001	0.033

^a^ DE miRNAs which were found in both tissues; ^b^ Log_2_FC: log2 fold-change; ^c^ Benjamini Hochberg false discovery rate (FDR) *p*-values.

**Table 3:** Correlations between miRNAs and adipogenic transcription factors or adipocyte genes in pigs with CLA.

	ssc-miR-21	ssc-miR-146b
**PPARγ**	–0.959*	–0.392
**C/EBPα**	–0.800	0.472
**FABP4**	0.863	–0.360
**FAS**	0.763	0.663
**FATP1**	0.581	0.242
**PGC-1α**	0.065	–0.055

The comparison between miRNAs and adipocyte gene expression was done by the Pearson chi-square test. Statistical significance (two-tailed). *p < 0.05.

## Supplementary Materials

The following can be found onlince at www.mdpi.com/2073-4425/8/10/271/s1, Figure S1: Small RNAs annotation; Figure S2: GO categories and distribution of 14 DE miRNA targets in pigs; Figure S3: The Wnt signalling pathway enriched by putative target genes of 14 DE miRNAs. Red boxes represent the target genes of miRNAs; Table S1: Composition of the basal diets for sows at different periods; Table S2: Datasets used in the study; Table S3: Primers used in this study; Table S4: The percentage of miRNA based on their length; Table S5: Predicted chromosomal positions, sequences, dominant arm, hairpin structure, minimum free energy, and counts of new miRNAs; Table S6: Information of known porcine miRNAs; Table S7: The read counts of all known porcine miRNAs; Table S8: Effects of CLA on body weight and fat deposition of pigs; Table S9: The information of log2 fold change for 14 DE miRNAs at each period; Table S10: The information of log2 fold change and FDR for new miRNAs; Table S11: KEGG pathways enriched for target genes of 14 DE miRNAs between control and CLA group. Supplemental File S1: RNA-seq data generated in this study.
