# Supplementary material for: Effects of Conjugated Linoleic Acid Supplementation on the Expression Profile of miRNAs in Porcine Adipose Tissue
Source: Genes (Basel). 2017 Oct 13;8(10):271. doi: 10.3390/genes8100271 (PMC5664121; doi:10.3390/genes8100271)
Supplement: Supplementary file 1 [file genes-08-00271-s001.zip › Supplementary Files/Fig S1.pdf]

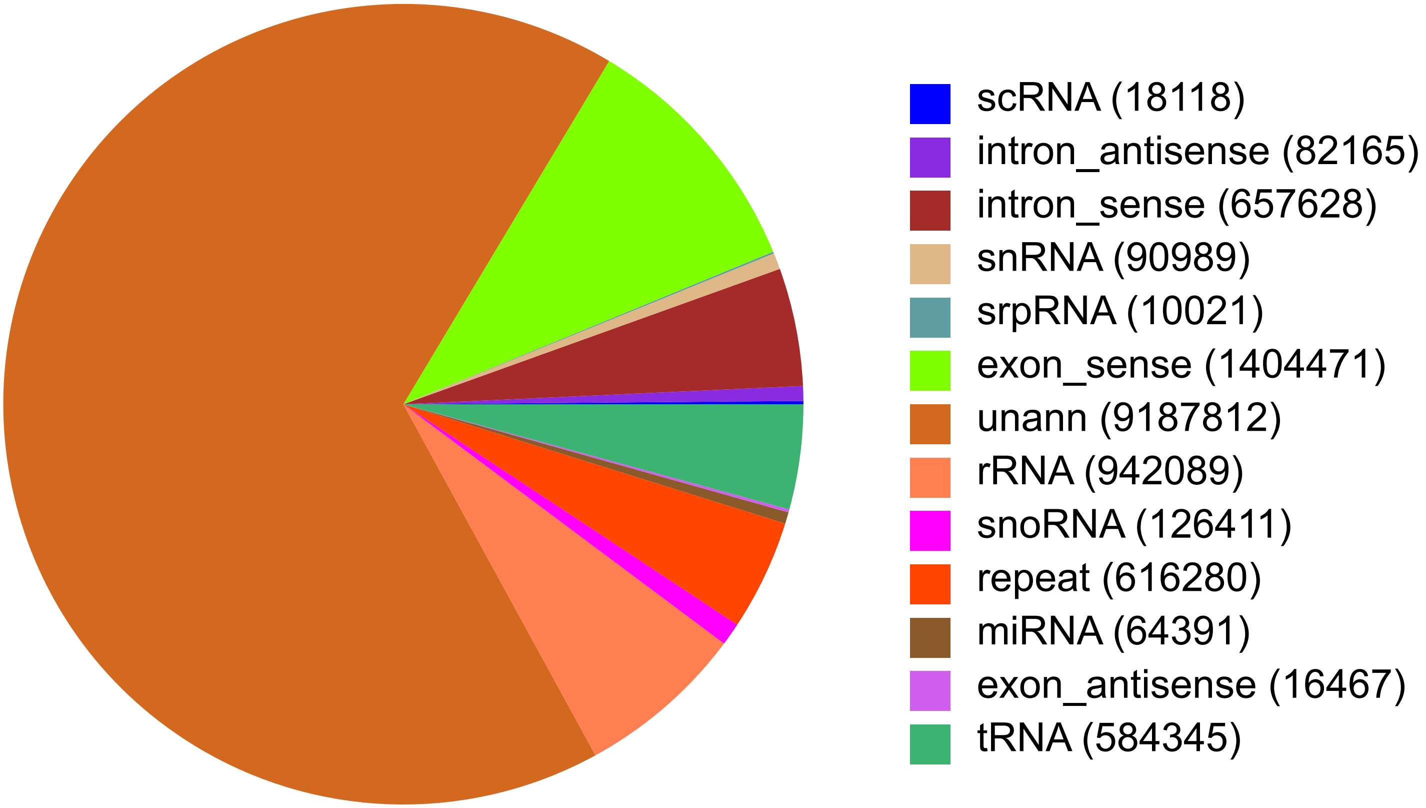
scRNA (18118)
intron_antisense (82165)
intron_sense (657628)
snRNA (90989)
srpRNA (10021)
exon_sense (1404471)
unann (9187812)
rRNA (942089)
snoRNA (126411)
repeat (616280)
miRNA (64391)
exon_antisense (16467)
tRNA (584345)
