# Supplementary material for: Effects of Conjugated Linoleic Acid Supplementation on the Expression Profile of miRNAs in Porcine Adipose Tissue
Source: Genes (Basel). 2017 Oct 13;8(10):271. doi: 10.3390/genes8100271 (PMC5664121; doi:10.3390/genes8100271)
Supplement: Supplementary file 1 [file genes-08-00271-s001.zip › Supplementary Files/Table S8. Effects of CLA on body weight and fat deposition of pigs.docx]

**Table S8.** Effects of CLA on body weight and fat deposition of pigs.

| **Items** | **Control** | **1.5% CLA** | ***P* value** |
| --- | --- | --- | --- |
| Body weight at slaughter/30day (kg) | 4.30±0.22 | 5.22±0.82 | 0.065 |
| Body weight at slaughter/90day (kg) | 78.90±0.47* | 20.66±1.00* | 0.038 |
| Body weight at slaughter/240day (kg) | 79.90±1.21** | 86.25±1.03** | 0.004 |
| Abdominal fat weight/240day (g) | 1366.80±137.88 | 1304.04±102.98 | 0.725 |
| [Thickness](javascript:void(0);) [of](javascript:void(0);) [back fat](javascript:void(0);)/240day (cm) | 5.42±0.07 | 5.37±0.09 | 0.649 |
| [Thickness](javascript:void(0);) [of](javascript:void(0);) waist fat/240day (cm) | 2.55±0.18 | 2.18±0.08 | 0.097 |
