# Supplementary figures and images for: Effects of Conjugated Linoleic Acid Supplementation on the Expression Profile of miRNAs in Porcine Adipose Tissue
Source: Genes (Basel). 2017 Oct 13;8(10):271. doi: 10.3390/genes8100271 (PMC5664121; doi:10.3390/genes8100271)

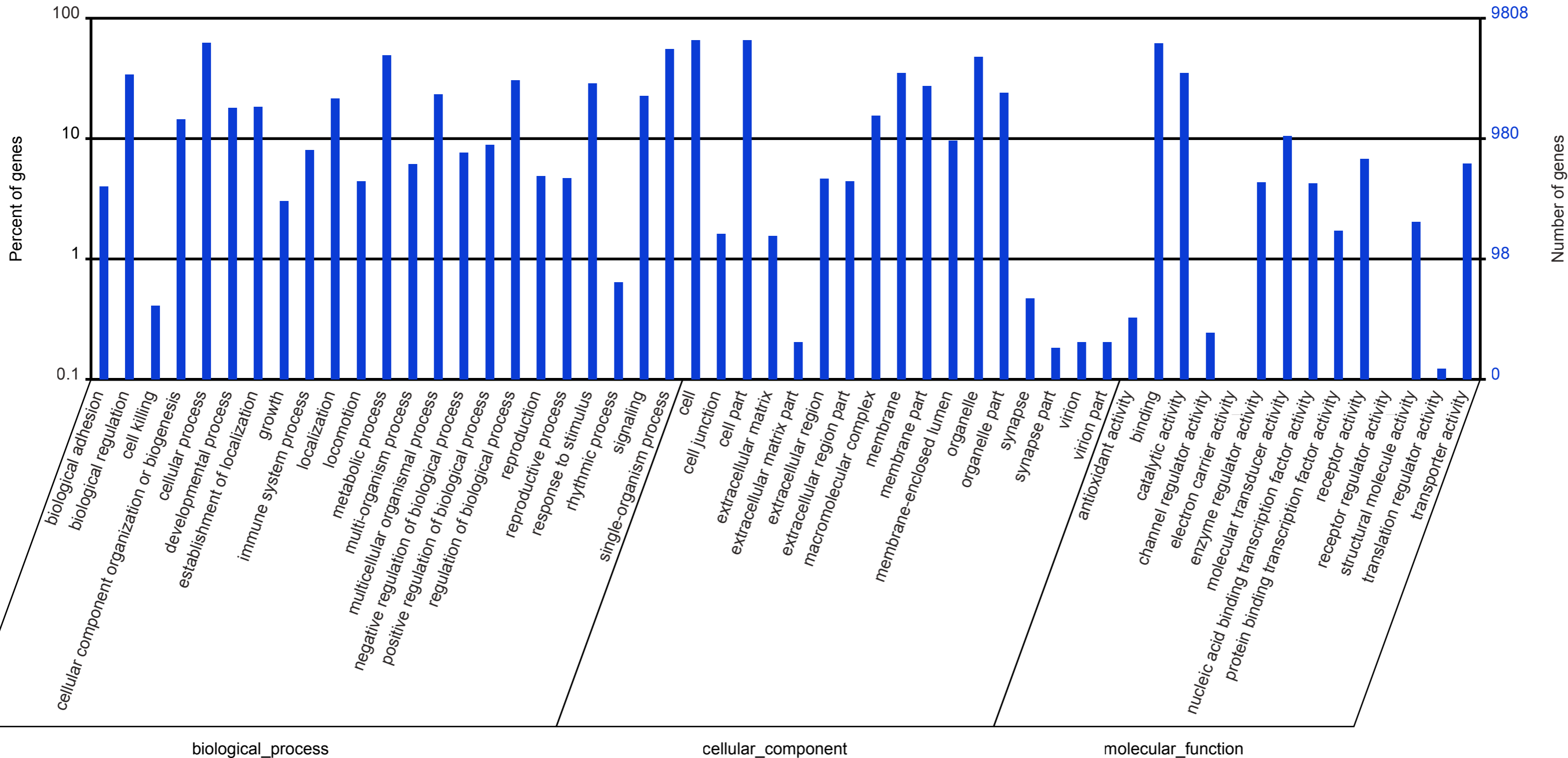

Supplement: Supplementary file 1 [file genes-08-00271-s001.zip › Supplementary Files/Fig S2.pdf]

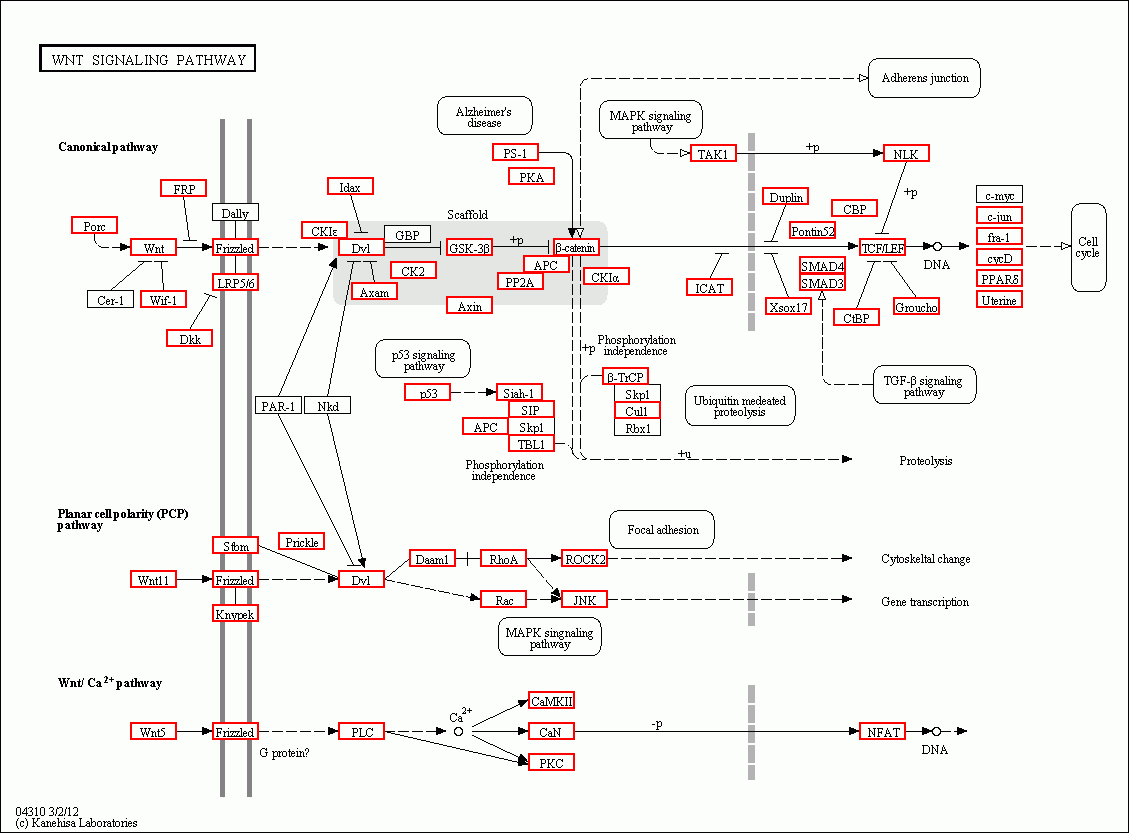

Supplement: Supplementary file 1 [file genes-08-00271-s001.zip › Supplementary Files/Fig S3.png]
