# Supplementary material for: Effects of Conjugated Linoleic Acid Supplementation on the Expression Profile of miRNAs in Porcine Adipose Tissue
Source: Genes (Basel). 2017 Oct 13;8(10):271. doi: 10.3390/genes8100271 (PMC5664121; doi:10.3390/genes8100271)
Supplement: Supplementary file 1 [file genes-08-00271-s001.zip › Supplementary Files/Table S1. Composition of the basal diets for sow at different periods.doc]

**Table S1.** Composition of the basal diets for sow at different periods.

(air-dry basis, %)

| **Items** | Early pregnancy | Late pregnancy |
| --- | --- | --- |
| **Ingredients (%)** |  |  |
| **Corn** | 59.49 | 62.26 |
| **Soybean meal** | 9.85 | 11.30 |
| **Wheat bran** | 25.64 | 20.71 |
| **Soybean oil** | 1.50 | 1.50 |
| **Limestone** | 0.61 | 1.15 |
| **Calcium hydrogen phosphate** | 1.18 | 1.20 |
| **NaCl** | 0.45 | 0.50 |
| **Lysine** | 0.00 | 0.10 |
| **Premix^a^** | 1.28 | 1.28 |
| **Total** | 100.00 | 100.00 |
| **Nutrient levels^b^** |  |  |
| **DE (MJ/kg)** | 12.68 | 12.84 |
| **CP** **(%)^b^** | 12.50 | 12.94 |
| **Ca (%)^b^** | 0.86 | 0.80 |
| **TP (%)** | 0.64 | 0.67 |
| **AP (%)** | 0.41 | 0.42 |
| **DLys (%)** | 0.55 | 0.65 |
| **DMet (%)** | 0.20 | 0.23 |
| **DMet+DCys (%)** | 0.41 | 0.46 |
| **DThr (%)** | 0.37 | 0.39 |
| **DTrp (%)** | 0.11 | 0.12 |

^a^Provided per kilogram of diet: Cu (CuSO_4_·5H_2_O) 80 mg, Fe (FeSO_4_·7H_2_O) 100 mg, Zn (ZnSO_4_·7H_2_O) 100 mg, Mn (MnSO_4_·H_2_O) 40 mg, Se (Na_2_SeO_3_) 0.3 mg, I (KI) 0.3 mg, VA 1 750 IU, VD3 200 IU, VE 11 IU, VK3 0.5 mg, niacin 20 mg, pantothenic acid 9 mg, folic acid 0.3 mg, VB1 1 mg, VB2 3 mg, VB6 1.5 mg, VB12 15 μg, biotin 0.05 mg, choline chloride 1.0 g, phytase 0.1 mg, anti-oxidant 0.5 mg. ^b^Measured value.
