# Supplementary material for: Effects of Conjugated Linoleic Acid Supplementation on the Expression Profile of miRNAs in Porcine Adipose Tissue
Source: Genes (Basel). 2017 Oct 13;8(10):271. doi: 10.3390/genes8100271 (PMC5664121; doi:10.3390/genes8100271)
Supplement: Supplementary file 1 [file genes-08-00271-s001.zip › Supplementary Files/Table S3. Primers used in this study.doc]

**Table S3.** Primers used in this study

| **Genes** | **Primer sequences (5ꞌ→3ꞌ)** |
| --- | --- |
| ssc-miR-1 | F: TGGAATGTAAAGAAGTATGTA |
| ssc-miR-21 | F: TAGCTTATCAGACTGATGTTGA |
| ssc-miR-145-5p | F: GTCCAGTTTTCCCAGGAATCCCTT |
| ssc-miR-146b | F: TGAGAACTGAATTCCATAGGC |
| ssc-miR-146a-5p | F: TGAGAACTGAATTCCATGGGTT |
| ssc-miR-206 | F:TGGAATGTAAGGAAGTGTGTGA |
| U6 | F: GCTTCGGCAGCACATATACT |
| PPARγ | F: TTGACCCAGAAAGCGAT |
|  | R: TATGGCACTTTGGTAGTCCTG |
| C/EBPα | F: AGAAGATCAACTGAGTGAACT |
|  | R: AGAGCTGAGAAACTAACGTG |
| FABP4 | F: AATTGGGCCAGGAATTTGAT |
|  | R: TCTTTCCATCCCACTTCTGC |
| FATP1 | F: CCACAGGCACCTTCAAGATT |
|  | R: GATCCAGAGGCAGGTAGTGG |
| FAS | F: CTACCTTGTGGATCACTGCATAGA |
|  | R: GGCGTCTCCTCCAAGTTCTG |
| PGC-1α | F: GCAGAAGAGCCGTCTCTACTTAAGA |
|  | R: TTTGCATGGTTCTGGGTACTGA |
| GAPDH | F: GAAGGTCGGAGTGAACGGAT |
|  | R: CATGGGTAGAATCATACTGGAACA |
